# Telepractice Treatment for Aphasia: Association Between Clinical Outcomes and Client Satisfaction

**DOI:** 10.1089/tmr.2020.0024

**Published:** 2021-03-24

**Authors:** Molly Jacobs, Patrick M. Briley, Xiangming Fang, Charles Ellis

**Affiliations:** ^1^ Department of Health Services and Information Management, East Carolina University, Greenville, North Carolina, USA.; ^2^ Communication Equity and Outcomes Laboratory, College of Allied Health Sciences, East Carolina University, Greenville, North Carolina, USA.; ^3^ Department of Communication Sciences and Disorders, College of Allied Health Sciences, East Carolina University, Greenville, North Carolina, USA.; ^4^ Department of Biostatistics, College of Allied Health Sciences, East Carolina University, Greenville, North Carolina, USA.

**Keywords:** aphasia, speech–language pathology, telepractice, satisfaction

## Abstract

**Introduction:** Health services research has demonstrated the association between patient satisfaction and treatment outcomes illustrating the importance of satisfaction in determining favorable treatment outcomes. Despite abundant evidence in the acute care setting, few researchers have explored these associations among patients receiving speech rehabilitation or therapeutic treatment particularly those receiving treatment through nontraditional delivery methods.

**Objective:** To examine the satisfaction with a community-based telepractice approach for treating aphasia among stroke survivors who reside in rural areas and assess potential correlations between satisfaction and patient outcomes.

**Methods:** In total, 22 adults with poststroke aphasia who resided in rural areas received comprehensive language-oriented treatment (LOT) for aphasia through community-based telepractice. Post-treatment satisfaction with the telepractice approach was assessed using the Client Satisfaction Questionnaire-8 (CSQ-8).

**Results:** After 12 sessions of LOT, Western Aphasia Battery-revised (WAB-R) aphasia quotients (AQs) improved on average 4.64 U. Mean scores on the CSQ-8 averaged 31.0/32.0, indicating a high level of satisfaction with the telepractice approach. In addition, each 1 U of improvement in patient satisfaction was associated with a 1.75 U increase in the WAB-R AQ.

**Conclusions:** Examination of post-treatment satisfaction indicated that satisfaction was highly predictive of effectiveness—a one-point increase in satisfaction was associated with a nearly two-point increase in WAB-R AQ. Results echo findings from acute care studies underscoring the importance of the patient experience in treatment efficacy.

## Background

In recent years, patient satisfaction has become increasingly important in health care. Not only is it an important outcome criterion, but it also provides a critical measure of health care quality.^1^ Satisfaction is a complex concept that relates to many factors, including lifestyle, past experiences, and future expectations, as well as individual values and those of society.^2^ Patient satisfaction may indicate the degree to which a patient feels they have received high-quality health care.^3^ It encompasses treatment itself as well as the treatment experience and delivery. Evidence suggests patient satisfaction and treatment outcomes are linked, thereby demonstrating overall patient satisfaction correlates with treatment outcomes.^4,5^

In the treatment of patients with chronic disease, Michie et al. found correlations between patient satisfaction, treatment compliance, and improvements in physical health.^6^ Similarly, Plewnia et al. noted a strong correlation in rehabilitation settings.^7^ Despite the evidence linking satisfaction to clinical outcomes, little is known about patient satisfaction among individuals receiving treatment for aphasia, a common communication disorder that occurs after stroke that results in significant communication difficulties. Persons with aphasia (PWA) experience problems with listening comprehension, verbal expression, reading, and writing, thereby limiting their communication interactions.^8^

PWA require substantial rehabilitation and there is an expansive literature related to aphasia rehabilitation and outcomes.^9^ However, few studies have examined PWA satisfaction with aphasia treatment. Tomkins et al. conducted a qualitative analysis of treatment satisfaction among 50 PWA receiving face-to-face treatment.^10^ They found seven factors that contributed to patient satisfaction, including (1) forming relationships; (2) manner and methods of service delivery; (3) information, communication, and knowledge; (4) structure and relevance of therapy; (5) organizational management; (6) individual support; and (7) positivity and improvement. In addition, PWA determined their level of satisfaction based on what information was provided, how much information was provided, how the information was provided, and their ability to engage in the communication exchange. Ultimately, the authors concluded that measures of satisfaction with aphasia treatment were influenced by both tangible factors and personal values.^10^

Although the use of rehabilitation to treat aphasia is a widely accepted practice, the use of telepractice approaches and other alternative delivery methods was unique in the United States until the onset of the novel coronavirus in 2019 (COVID-19). The American Speech-Language-Hearing Association (ASHA) defines “telepractice” as “the application of telecommunications technology to the delivery of speech–language pathology and audiology professional services at a distance by linking clinician to client or clinician to clinician for assessment, intervention, and/or consultation.”^11^ ASHA designated the term “telepractice” rather than telemedicine or telehealth to minimize the misperception that such services are only utilized in health care settings.^11^ Consequently, with the emergence of telepractice approaches to aphasia treatment, there is a need to understand the impact of this shift in practice patterns from primarily face-to-face to telepractice approaches. The rapid spread of COVID-19 demanded a dramatic and urgent shift to the use of remote forms of treatment delivery for aphasia.^12–14^ Similarly, there was a need to understand the impact of this shift in practice on patient satisfaction.

Tousignant et al. previously examined patient satisfaction after telepractice for poststroke aphasia using a sample of 20 patients receiving 3 weeks of telepractice treatment in Canada.^15^ They measured satisfaction with a French adaptation of the Telemedicine Satisfaction Questionnaire after the completion of a pragmatic approach to aphasia treatment. They showed that patients with poststroke aphasia receiving speech teletherapy were very satisfied with this service delivery method.

A second study by Pitt and colleagues completed in Australia also found high patient satisfaction after a group-based 2-week intensive constraint-induced language therapy delivered through web-based videoconferencing program.^16^ Finally, aphasia telepractice studies by Choi et al. in South Korea^17^ and by Fink et al.^18^ in the United States both included measures of satisfaction but did not focus on satisfaction as a primary determinant nor include a standardized measure of satisfaction. To date, studies in the United States have not specifically emphasized satisfaction with telepractice approaches for treating aphasia.

This study was designed to examine the relationship between patient satisfaction and treatment outcomes after comprehensive treatment (addressing verbal expression and auditory comprehension) for aphasia using a community-based sample of PWA receiving treatment through a telepractice approach. We were interested in understanding patient satisfaction with a community-based telepractice approach for treating aphasia among stroke survivors who reside in rural areas and assess potential correlations between satisfaction and patient outcomes. Community-based treatment allows clients to receive treatment close to their homes from providers that may not be available in their local communities.

**FIG. 1.**
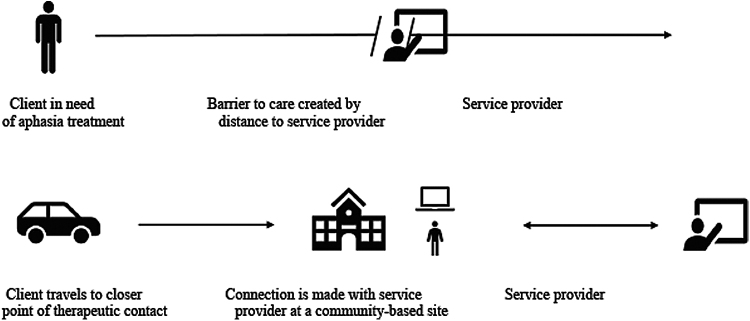
Point of therapeutic contact improved through utilization of telepractice at a community-based site.

Increased accessibility of care increases the likelihood that the estimated 2.2–2.5 million PWA living in the United States will receive the needed treatment for the condition.^19^ Thus, access to care is improved without extraneous travel requirement or contact with individuals outside of one's own local area.^20^ For PWA living in rural areas specifically, such an approach can improve access to care by (1) reducing drive time to the point of therapeutic contact that is typically in more urban areas and (2) increasing the likelihood of service provision (Fig. 1).

Finally, it is also possible that community-based telepractice approaches for PWA offer greater opportunities for community reintegration that are frequently absent among PWA.^21–23^ Therefore, the objective of the study was to examine the satisfaction with a community-based telepractice approach for treating aphasia among stroke survivors who reside in rural areas and assess potential correlations between satisfaction and patient outcomes.

## Methods

### Study participants

Twenty-two PWA were enrolled in the study. The study inclusion criteria included (1) communication skills necessary to complete telepractice sessions, (2) ability to answer 70% of yes/no questions on the Western Aphasia Battery-revised (WAB-R),^24^ and (3) at least 18 years of age. The WAB-R yes/no questions provided the key metric for inclusion in the study. Inclusion criteria based on the WAB-R AQ would have excluded individuals with significant expressive language and motor speech deficits who would have the comprehension skills to engage in the treatment approach.^25^ Individuals who scored >93.8 on the WAB-R AQ (cutoff score for aphasia) were included in the study if they (1) had a previously confirmed diagnosis of aphasia and (2) reported persisting word retrieval issues.^26^ Exclusion criteria included (1) participants who had dementia, a progressive neurological or cognitive disorder or a severe communication disorder that would prevent participation and (2) participants who were unable to drive or have a family member or caregiver who was willing to participate in the study to ensure transportation to the community-based site. Participants also had to agree to at least 6 weeks of treatment, two times per week.

### Recruitment

All potential participants resided in rural counties in eastern North Carolina, defined as population density <80 people per square mile.^27^ Participants were recruited locally from local inpatient and outpatient clinics at a local hospital, Veterans Administration health care clinic, and community senior centers. Speech–language pathologists at the aforementioned clinics were notified at the onset of the study, and periodically, about the goals and objectives of the study. The participants treating (and referring) clinician provided them with information about the study, as outlined in the institutional review board (IRB) approval. Potential participants then contacted the study's principal investigator and scheduled a face-to-face meeting at the last author's (C.E.) laboratory to evaluate their ability to meet inclusion criteria and enroll in the study. Language evaluations for inclusion were completed by C.E. or graduate students supervised by C.E. Those who met inclusion criteria provided written consent and scheduled their first appointment at the designated community location.

### Telepractice platform

The aphasia rehabilitation treatment was delivered by Lenovo T570 ThinkPad laptop computers with 15″ monitors through Webex™, a cloud-based videoconferencing program that allows real-time exchange of video and audio for individuals at a distant location through a secure internet connection. Webex offers both full screen and side-by-side sharing views, which allowed the research team and patient collaborative sharing of treatment materials and other documents required for the administration of the aphasia treatment. The treating clinician for the project provided the aphasia telepractice from a medical campus aphasia laboratory site, and the PWA received the treatment at a remote community-based site (local school or senior center). Student facilitators (undergraduate and graduate students enrolled in a Communication Sciences and Disorders program) were available at the remote treatment site to set up the equipment and assist the participants.

### Aphasia treatment details

Participants were scheduled to complete 12 treatment sessions for a 6-week period. The provider, a certified speech–language pathologist, had ∼30 years' experience. Participants received their telepractice during individual sessions in a quiet room with the student facilitators present. Some caregivers were also present during the session but did not participate. The general aphasia treatment used through telepractice was the language-oriented treatment (LOT).^28,29^ The LOT was designed to address a range of language issues among PWA. The LOT approach is highly structured that is advantageous for measuring fidelity and replication. Treatment targets for comprehension included improving access to word meanings and changing the individual's communication environment to support auditory comprehension. Targets for expression included spoken output with written letters, repetition, and reading aloud. Whereas LOT facilitated the general approach to treatment, participants required individualized programs to address aphasia-related deficits.

 Consequently, the team utilized a range of evidence-based treatments designed to improve overall communication including semantic feature analysis,^30^ verb network strengthening treatment,^31^ and combined aphasia and apraxia of speech treatment.^32^ Similarly, PWA frequently have coexisting motor speech production disorders (apraxia and dysarthria). For those individuals, we addressed those deficits using the Mayo Clinic approach for treatment of motor speech disorders that is a systematic treatment approach.^33^

### Outcome measures

Aphasia impairment was measured pre- and post-treatment using the WAB-R AQ. Impairment change scores were calculated based on pre- and post-treatment WAB-R AQs.^24^ Treatment satisfaction was assessed using the eight-item Client Satisfaction Questionnaire (CSQ-8).^34^ All eight questions assign numeric scores ranging from 1 (low satisfaction) to 4 (high satisfaction) with a cumulative score of 32.

### Statistical analysis

Descriptive statistics were calculated for all demographic characteristics and treatment outcomes using R version 3.6.4. The association between treatment effectiveness and satisfaction was assessed using Bayesian random effects models. Bayesian analytics allow prior information to be incorporated into covariate specific probability distributions. It also allows for the estimation of a large number of random variance components without the large data requirements needed for frequentists methods.

## Results

### Sample demographics

The mean age of the sample was 61 (standard deviation [SD] 14.2) years and a mean education of 14.0 (SD 2.3) years (Table 1). The sample was on average 42.5 months (SD 15.5) poststroke onset with participants ranging from 1 to 288 months postonset.

**Table 1.** Patient Demographic and Outcomes Data for Telepractice Study

	Mean (SD)	Median	Range
Pretreatment			
Age (years)	61.0 (14.2)	64.5	33–96
Education (years)	14.0 (2.3)	2.39	9–20
Time poststroke onset (months)	42.5 (15.5)	15.5	1–288
WAB-AQ initial	73.9 (23.4)	83.7	26.2–99.0
Posttreatment			
WAB AQ change	4.64 (6.53)	4.15	−8.1 to 14.5
CSQ-8 satisfaction score	31.0 (1.4)	32.0	28–32

AQ, aphasia quotient; CSQ-8, Client Satisfaction Questionnaire-8; SD, standard deviation; WAB, Western Aphasia Battery.

### Aphasia impairment

Baseline WAB-R AQ was 74.9 (SD 23.4) (Table 2). WAB-R AQ scores increased on average 4.64 points. Change scores ranged from −8.1 to 14.5.

**Table 2.** Bayesian Mixed Effects Estimation Results

	Estimate	Estimated error	95% CI lower	95% CI upper
Intercept	−47.4	40.75	−129.74	30.9
CSQ-8	1.8	1.38	−0.97	4.6
TPO months	−0.02	0.04	−0.11	0.05
Age	−0.1	0.13	−0.36	0.17
African American	1.42	3.84	−5.84	9.37
Broca's aphasia	4.03	4.25	−4.04	12.46
Conduction aphasia	8.48	5.3	−2.72	18.85

	Estimate	Estimated error	Quant. 1	Quant.3
*R*^2^	0.41	0.08	0.19	0.56
Reference category: anomia				
Dependent variable: ΔWAB-R AQ = WAB-R AQ final−WAB-R AQ initial				

CI, confidence interval; TPO, time postonset; WAB-R, WAB-revised.

### Satisfaction

Scores on the CSQ-8, for the 22 participants who completed the study, averaged 31.0 (SD 1.4) out of a maximum score of 32. Participant scores ranged from 28 to 32. Scores on the CSQ-8 indicated a high level of satisfaction with the telepractice treatment approach.

### Patient satisfaction as a function of aphasia impairment

Each 1 U of improvement in patient satisfaction was associated with a 1.75 (standard error [SE] = 1.26) U increase in the WAB-R AQ. This increase was substantially larger than the change in WAB-R AQ observed from age (−0.14, SE = 0.13), race (0.77, SE = 3.67), or months postonset (−0.03, SE = 0.05). Type-specific fixed effects and individual-specific intercepts accounted for condition and individual-specific variations in effectiveness. Therefore, the effect of satisfaction is observed even after accounting for variation by type of aphasia.

## Discussion

This study examined patient satisfaction in community-based telepractice treatment for aphasia and tested the relationship between patient satisfaction with community-based aphasia treatment and treatment outcomes. The findings of this study are significant on two levels. First, evidence suggests that ∼20% of stroke survivors experience aphasia.^35^ Telepractice provides an additional mechanism to treat this growing population particularly when traditional face-to-face treatments may not be feasible. Second, the recent COVID-19 pandemic forced many providers to abruptly transition to remote treatment and this study offers practitioners clear evidence about the efficacy, feasibility, and satisfaction of aphasia treatment telepractice and in particular to address the treatment needs of rural residents.

PWA CSQ-8 scores ranged from 28 to 32 indicating high satisfaction with the telepractice approach. These findings align with those of Tousignant and colleagues who also noted high levels of patient satisfaction in a 3-week telepractice intervention for chronic aphasia.^15^ More importantly, this study showed that patient satisfaction was closely tied to clinical improvement—change in WAB-R AQ. Patients improved between 0.2 and 12.4 U with an average of 4.64 U. Typically, a five-point improvement on WAR-AQ is considered a clinically relevant change.^36^ Improvement directly corresponded with satisfaction such that for each one-point increase in satisfaction, aphasia impairment improved by nearly two points.

The improved clinical outcomes in relationship with satisfaction are not entirely a surprise. Previous studies have shown that telepractice approaches can improve a range of skills after aphasia, including naming, fluency, and auditory comprehension while reducing overall level of aphasia impairment.^17,37^ Studies also indicate that a range of different telepractice approaches can be successful in improving aphasia outcomes such computer/tablet-based and in-home telepractice.^38–42^ More importantly, it is well established that telepractice approaches can have other positive outcomes beyond communication such as improved overall quality of life.^43,44^

Despite some participants' limited technological experience, the incorporation of technology did not hinder satisfaction with the approach. PWA expressed specific satisfaction with two treatment aspects. First, nearly all patients commented that seeing the therapist and stimuli simultaneously was advantageous compared with traditional face-to-face treatment requiring clients to look down at the stimuli, thus limiting their ability to view the therapist's feedback. Second, participants were highly satisfied with the sound and video quality of the computer connection. With regard to these aspects, PWA expressed higher satisfaction with telepractice than face-to-face speech–language pathology service treatments for aphasia. More importantly, the approach utilized in this study allowed individuals to receive treatment within their local areas.

Despite these interesting findings, there are some limitations. Many of the PWA were many years poststroke onset. Thus, it was unclear whether their satisfaction was primarily influenced by the novel telepractice approach or their ability to receive much needed services. In addition, the PWA in this study included a wide range of severity levels with large variation in functional ability. Given the presence of aphasia, responses to the outcome scales could have been subject to misunderstanding or misinterpretation. Similarly, the treatment was primarily provided by one therapist, thus creating the possibility of bias in the satisfaction reports. Finally, the scores achieved on the satisfaction scale were all high and within a few points, thus not allowing the investigators to determine any significant distinction between the participants. Despite these potential limitations, the data inarguably show high level of both communication ability and treatment satisfaction.

## Conclusions

Using a community-based telepractice treatment for aphasia, this study tested the association between treatment outcomes and patient satisfaction—a hypothesis frequently proven in acute and primary care research. In addition to a significant improvement in fluency, patients were highly satisfied with treatment. Satisfaction level was highly predictive of treatment outcome, validating findings from other health care sectors. Results underscore the important role that patient experience plays in treatment efficacy. Future studies will be needed to a larger sample to further examine spread of the satisfaction measure scores. Future studies must also be designed to compare the satisfaction of telepractice for aphasia with in-person treatment for aphasia.

## Abbreviations Used

AQ: aphasia quotient
ASHA: American Speech-Language-Hearing Association
CI: confidence interval
COVID-19: coronavirus disease 2019
CSQ-8: Client Satisfaction Questionnaire-8
IRB: institutional review board
LOT: language-oriented treatment
PWA: persons with aphasia
SD: standard deviation
SE: standard error
TPO: time postonset
WAB: Western Aphasia Battery
WAB-R: WAB-revised
